# Requirement of NF-kappa B Activation in Different Mice Brain Areas during Long-Term Memory Consolidation in Two Contextual One-Trial Tasks with Opposing Valences

**DOI:** 10.3389/fnmol.2017.00104

**Published:** 2017-04-07

**Authors:** Angeles Salles, Maria del C. Krawczyk, Mariano Blake, Arturo Romano, Mariano M. Boccia, Ramiro Freudenthal

**Affiliations:** ^1^Laboratorio de Neurobiología de la Memoria, Departamento de Fisiología y Biología Molecular y Celular, Facultad de Ciencias Exactas y Naturales, Universidad de Buenos AiresBuenos Aires, Argentina; ^2^Instituto de Fisiología, Biología Molecular y Neurociencias (IFIBYNE), CONICET-Universidad de Buenos AiresBuenos Aires, Argentina; ^3^Laboratorio de Neurofarmacología de los Procesos de Memoria, Cátedra de Farmacología, Fac. Farmacia y Bioquímica, Universidad de Buenos Aires/CONICETBuenos Aires, Argentina; ^4^ Departamento de Fisiología, Instituto de Fisiología y Biofísica Bernardo Houssay (IFIBIO), Facultad de Medicina, Universidad de Buenos Aires, CONICETBuenos Aires, Argentina

**Keywords:** NF-kappa B, inhibitory avoidance learning, hippocampus, amygdala, nucleus accumbens, one-trial learning

## Abstract

NF-kappa B is a transcription factor whose activation has been shown to be necessary for long-term memory consolidation in several species. NF-kappa B is activated and translocates to the nucleus of cells in a specific temporal window during consolidation. Our work focuses on a one trial learning tasks associated to the inhibitory avoidance (IA) setting. Mice were trained either receiving or not a footshock when entering a dark compartment (aversive vs. appetitive learning). Regardless of training condition (appetitive or aversive), latencies to step-through during testing were significantly different to those measured during training. Additionally, these testing latencies were also different from those of a control group that only received a shock unrelated to context. Moreover, nuclear NF-kappa B DNA-binding activity was augmented in the aversive and the appetitive tasks when compared with control and naïve animals. NF-kappa B inhibition by Sulfasalazine injected either in the Hippocampus, Amygdala or Nucleus accumbens immediately after training was able to impair retention in both training versions. Our results suggest that NF-kappa B is a critical molecular step, in different brain areas on memory consolidation. This was the case for both the IA task and also the modified version of the same task where the footshock was omitted during training. This work aims to further investigate how appetitive and aversive memories are consolidated.

## Introduction

The ability to store long-term memories for periods ranging from days to a lifetime is one of the most important characteristics of the brain that allows adapting and predicting events in a changing environment. Long-term memory consolidation has proven to be dependent on *de novo* transcriptional activity regulated by transcription factors (Alberini and Kandel, 2015). Among the transcription factors involved in the regulation of gene expression during consolidation, NF-kappa B has been found to be activated and necessary during the consolidation of appetitive and aversive memories in several paradigms (Meffert et al., 2003; de la Fuente et al., 2015). In its canonical pathway, NF-kappa B is a dimer composed by p65 and p50 that in the cytoplasm is in association with I kappa B its inhibitor. Upon neuronal activity convergent paths result in I kappa B phosphorylation and degradation, allowing NF-kappa B to translocate to the nucleus. In our laboratory, the nuclear activation of NF-kappa B has been described during the long-term inhibitory avoidance (IA) memory consolidation, in the mouse hippocampus and was specific for the association between the shock and the context (Boccia et al., 2007). Inhibiting hippocampal NF-kappa B activation with a decoy κb consensus sequence or Sulfasalazine has proven to induce amnesia only when administered during the consolidation time window (Merlo et al., 2005). Sulfasalazine specifically impedes NF-kappa B activation, inhibiting I kappa B kinase activity by blocking its ATP binding site and not allowing NF-kappa B to translocate to the nucleus (Wahl et al., 1998). The Sulfasalazine effect is not mediated by its effect on cyclooxygenase (Merlo et al., 2002).

Two kinds of memories with opposing valences could be the result of adverse experiences depending on timing of stimuli. On one hand, “negative” memories are induced when stimuli is preceding the adverse experience. On the other hand, “positive” memories are induced when stimuli is experienced at the moment of “relief” (Niewalda et al., 2015). Entering a dark compartment for a mouse after being exposed to an illuminated platform could be considered as a positive and relief experience. An appetitive experience can be defined as one that increases the likelihood of satisfying a particular animal’s need (such as finding shelter) or as a state of quiescence whose prerequisite is the absence of a disturbing stimulus (Lorenz, 1981). In this work, this definition will be used to refer to an appetitive experience. These kind of aversive and relief memories could be found in phylogenetically distant species such as fly, rats, mice and humans (Andreatta et al., 2012; Vogt et al., 2015).

Here we use two tasks associated to the IA setting. The two tasks share contextual information and operant behavior, but have a different reinforcement when entering the dark compartment. The animals that receive the electric stimulus form a “negative” memory that expresses as longer latencies to step-through on testing day (Boccia et al., 2005). The animals that do not receive the stimulus form a “positive” memory and show shorter latencies to step-through when tested. This last task has similarities with a modified version of the Barnes maze for mice (Koopmans et al., 2003), with the difference that the protocol presented here is a one trial learning task. In the modified version of the Barnes maze the mice step-through one of 12 entrances in a wall that surround an illuminated arena. Both the Barnes maze and the Un-shocked task used here have the benefit that no strong aversive stimuli or deprivation procedures have to be used for the animal to respond. These tasks take advantage of the natural preference of rodents for dark environments (Pompl et al., 1999).

In the present work we evaluate the effect of NF-kappa B inhibition on brain areas that have been previously involved in the processing of the context and valence information in during memory consolidation (Boccia et al., 2007; Smith and Bulkin, 2014; Namburi et al., 2016); and where the transcription factor has been described (Ang et al., 2001; Freudenthal et al., 2004; Si et al., 2012). The present work shows for the first time that the consolidation of appetitive and aversive memories of a contextual one-trial task both depend on the activation of NF-kappa B in three different brain structures: Hippocampus, Amygdala and Nucleus accumbens.

## Experimental Procedures

### Animals

The experiments were carried out following the National Institute of Health Guide for the Care and Use of Laboratory Animals (NIH publication no. 80-23/96) and the protocol was approved by Comisión institucional para el cuidado y uso de animales de laboratorio (CICUAL N°0071). CF-1 male mice from our own breeding stock were used (*Mus musculus*; age 40–50 days; weight 25–30 g). Mice were kept in a lodging room maintained at 21–23°C on a 12-h light–dark cycle (lights on at 06.00 h), with *ad libitum* access to dry food and tap water. All efforts were made to reduce the number of animals used and ameliorate animal suffering.

### Apparatus and Behavioral Procedure

IA behavior was studied in a one-trial learning, step-through type situation (1), which utilizes the natural preference of mice for dark environments. The apparatus consists of a dark compartment (20 × 20 × 15 cm) with a stainless-steel grid floor and a small (5 × 5 cm) illuminated and elevated platform attached to its front center. Mice were not habituated to the dark compartment before the learning trial. All mice were trained between 8 am and 10 am. Three groups of animals were used for the experiments, Shocked (S), Un-shocked (U) and Immediate shock group (Si). During training, S animals were placed on the illuminated platform and received a footshock as it stepped into the dark compartment. The footshock-training conditions were 1.2 mA, 50 Hz, 1 s. The U animals did not receive a footshock when entering the dark compartment. For all groups retention was evidenced by median scores when entering the dark compartment during testing, 48 h post training. Si animals are placed directly inside of the dark compartment and immediately receive the inescapable footshock after which they are returned to their home cage. For assessment of NF-kappa B binding activity a Naïve (N) group of animals was included. The (N) group mice were housed in the same conditions as that of the experimental groups but do not experience the experimental setting, and this group was included in order to estimate basal levels.

### Surgical Procedures and Drugs

Mice were implanted under deep anesthesia (0.1 ml of ketamine 80% and xylazine 20%) with 23 gauge guide cannulae 5 days before training. For the hippocampus, coordinates were anterior-posterior axis (AP), −1.9; lateral axis (L), ±1.2; and dorso-ventral axis (DV), −1.2; for amygdala coordinates were AP, −1.5; L, ±3; and DV, −3.5; for nucleus accumbens coordinates were AP, +1.6; L, ±1; and DV, −3.1; and for primary somatosensory cortex (forelimb region) coordinates were AP, + 0.5; L, ±2.5; and DV, −1; from Bregma in accordance with the atlas of Paxinos and Franklin (2013). Guide cannulae were fixed to the skull with dental acrylic. In all cases injections were performed bilaterally and immediately after training session. The injection cannula was inserted into the guide cannula with its tip extending beyond the guide by 1 mm to reach the different areas except for the primary somatosensory cortex (forelimb region) where the injection cannula extended only 0.5 mm. The injections were administered during 30 s and operated by hand. The injection cannula was removed after 60 s to avoid reflux and to allow the diffusion of drugs. The volume of each infusion was 0.5 μl/side. After behavioral procedures histological examination of cannulae placements was performed. For this purpose, animals were injected with India ink and decapitated, brains were placed in 4% paraformaldehyde for 1 day followed by 30% sucrose for an additional 24 h and after this treatment brains were sliced using a vibratome. Finally, cannulae placement was verified with a magnifying glass. Only data from animals with cannulae located in the intended areas were included in the analysis.

Sulfasalazine (2-4-Hydroxy((4-((2-pyridinilamino)sulfonyl)phenyl)azo)-benzoic acid) (Sigma, St. Louis, MO, USA) was injected in a solution (2 μg/μl) 100% DMSO, vehicle was a solution containing 100% DMSO (Veh). The dose used has been proven to specifically inhibit NF-kappa B, and this has been shown in previous publications (Freudenthal et al., 2005; Boccia et al., 2007).

### Nuclear Extracts

The mice were killed by cervical dislocation, N animals were sacrificed at the same time as S, U and Si animals for each experiment. The brains were rapidly removed, and the hippocampus was dissected according to the method of Iversen and Glowinski (1966). To obtain nuclear extracts, tissues were homogenized in 250 μl of hypotonic buffer A (10 mM HEPES, pH 7.9, 10 mM KCl, 1.5 mM MgCl_2_, 1 mM DTT, 1 g/ml pepstatin A, 10 g/ml leupeptin, 0.5 mM PMSF, and 10 g/ml aprotinin) with eight strokes in a Dounce homogenizer, type B pestle. The homogenate was centrifuged for 15 min at 1000 *g* and the supernatant was discarded. The pellet was resuspended in 30 μl of hypertonic buffer B (20 mM HEPES, pH 7.9, 800 mM KCl, 1.5 mM MgCl_2_, 0.4 mM EDTA, 0.5 mM DTT, 50% glycerol, 1 g/ml pepstatin A, 10 g/ml leupeptin, 0.5 mM PMSF, and 10 g/ml aprotinin) and incubated for 15 min on ice. A centrifugation for 15 min at 10,000 *g* was then performed. The supernatant (nuclear extract) was stored at −80°C until used. The entire extraction protocol was performed at 4°C.

### Determination of DNA-Binding Activity

κB DNA binding activity in nuclear fractions was assessed using electrophoretic mobility shift assay (EMSA). Double-stranded oligonucleotide DNA containing the NF-kappa B binding site (5′-AGTTGAGGGGACTTTCCCAGGC-3′ binding site in bold) was prepared by annealing two different length single stranded oligonucleotides (5′-AGTTGAGGGGACTTT-3′ and 5′-GCCTGGGAAAGTCCCCTCAACT-3′ custom made, Integrated DNA Technologies) and completing the overhanging end with the Klenow (Promega) enzyme protocol. For this, 5 mM of each dGTP, dATP, dTTP and labeled dCTP^32^ were incubated with 2 picomoles of double strand oligonucleotide with the overhanging end, the 2.5 units of Klenow enzyme and Buffer for 20 min at room temperature. The reaction was stopped with 2 μl of 200 mM EDTA. The DNA was precipitated overnight following the addition of sodium acetate and ethanol to eliminate free dCTP^32^, next day the oligonucleotide was centrifuged and resuspended in 150 μl of water. DNA-protein binding was carried out in 20 μl of Hepes, 20 mm, pH 7.9; KCl, 120 mm; EDTA, 0.4 mm; DTT, 0.5 mm; glycerol, 25%; 0.3 μg of poly dIdC and 10 μg of protein extract. Samples were incubated for 40 min at 0°C and then 1 μl of labeled oligonucleotide probe was added to each sample followed by incubation for a further 40 min at 0°C. The reaction mixture was electrophoresed on a 6% non-denaturing polyacrilamide gel in 0.25 × TBE (in mM: Tris, 22.3; boric acid, 22.3; and EDTA 0.5) for 2 h at 170 V. The gel was vacuum-dried and exposed overnight to Hyperfilm MP (Amersham). All measures were made with exposures within the linear range of the film. Images were digitized by means of a scanner (Umax PowerLook III). The relative optical density (ROD) of the specific band was estimated using NIH ImageJ 1.45s software. Protein contents of the extracts were measured in duplicate by the BCA method (Pierce—Thermo Scientific) and checked for quality and quantity by comparing pattern intensities of Lamin B1 (Santa Cruz Biotechnology) in SDS-PAGE. Western Blots were done as previously described in Salles et al. (2015).

### Data Analysis

ROD values for each group were related to the mean ROD values of the N group. One-way ANOVA or Kruskal-Wallis test were used to estimate general significance. Differences between groups were assessed by *post hoc* Fisher LSD test. Mann-Whitney non-parametric pairwise comparisons were made for comparing between same training protocol and different drug/vehicle groups, or same vehicle and different training protocol. Bonferroni’s correction was made to determine statistical significance (i.e., for hippocampus *p* < 0.0033). Wilcoxon paired non-parametric test using a free statistical software package. The parametric tests used for the biochemical data follow all assumptions of normality and homogeneity of variance. The statistical tests for the behavioral experiments chosen are non-parametric due to the fact that the latencies to step-through do not show a normal distribution because the animals are removed from the platform after 5 min of testing. *p* < 0.05 was considered as statistically significant.

## Results

### Both Shock and Un-Shocked Animals Show Retention 48 h Post-Training

**Figure 1:**
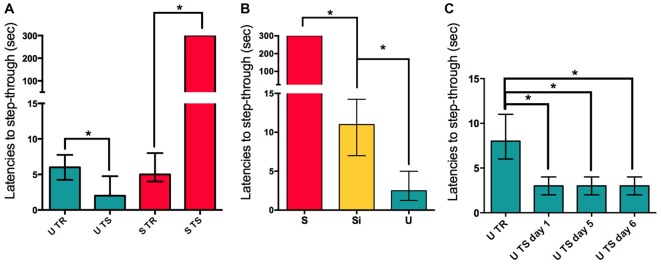
**Aversive and appetitive training causes long-term memory consolidation. (A)** Retention is evidenced in S and U groups by comparing latencies to step-through during training (TR) for U and S groups compared to the latencies to step-through for the same groups of animals during testing (TS). **(B)** Latencies to step-through during TS for S, U and Si groups, statistical comparisons are made against the Si group. **(C)** Latencies to step-through for the U animals during training and consecutive re-exposure days. Bars show medians with interquartile ranges. *P < 0.01.

The first experiment showed that both Shocked (S, *n* = 15) and Un-shocked (U, *n* = 16) animals changed their behavioral response in the testing session (48 h post-training) in comparison to the training session (Wilcoxon *W* = 120 *p* < 0.01 for S; and Wilcoxon *W* = −101.0 *p* < 0.01 U vs. training day for each group; Figure 1A). The behavioral changes observed for each group differed from each other in the fact that latencies to step-through during testing increased in S while they decreased in U animals. To contrast S and U groups with animals having a minimal operational response during training, we included the immediate shock group. The Immediate Shock (Si) animals are placed into the dark compartment directly from the top and have no access to the illuminated platform; and immediately receive a foot shock before being returned to their home cage. During the testing session, Si animals show latencies similar to naïve animals when placed on the illuminated platform (Median = 11 s; *n* = 8). S animals, as previously described, show significantly higher latencies to step-through into the dark compartment, evidencing retention (Mann Whitney *U* = 0.0; *p* < 0.01; Median = 300 s; *n* = 8), while U animals show significantly shorter latencies when entering the dark compartment (Mann Whitney *U* = 0.0; *p* < 0.01; Median = 2.5 s; *n* = 8), both when compared to Si animals (Kruskal-Wallis statistic *H*_(2,21)_ = 21.08, *p* < 0.01; Figure 1B). This experiment shows that while Si animals have latencies in the range of Naïve animals, S animals learn to avoid entering the dark compartment and U animals learn to enter it even faster. Interestingly, U animals continue to show low latencies to step-through when tested in consecutive days for 6 days, evidencing also, a limit to the speed at which they can enter the dark compartment (ANOVA Friedman statistic = 21.86; *p* < 0.01; Median for TR day = 8; Median for day 1 = 3; Median for day 5 = 3; Median for day 6 = 3; *n* = 20; Figure 1C). This data supports the idea that both groups of animals are forming contextual memories.

### NF-kappa B is Activated in the Nucleus of Hippocampal Cells 45 min Post-Training Both in S and U Animals but Not in Si Animals

**Figure 2:**
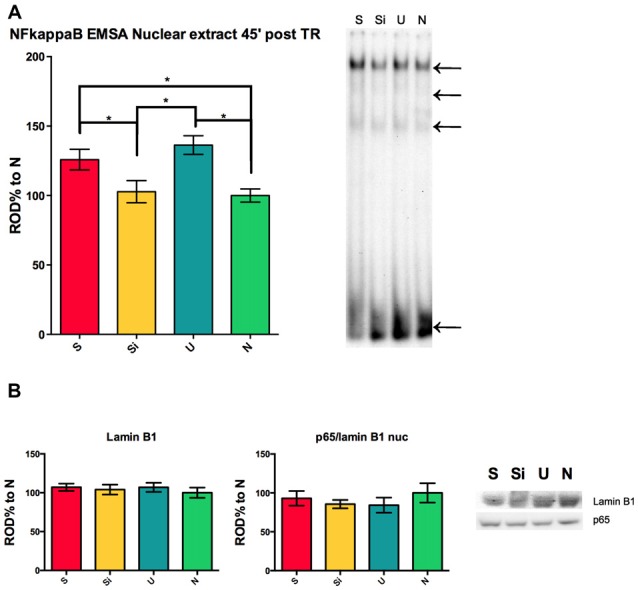
**NF-kappa B activity in the hippocampus during long-term memory consolidation of aversive and appetitive tasks. (A)** Left panel shows relative optical density (ROD%) for electrophoretic mobility shift assay (EMSA) NF-kappa B activity 45 min post-training for S, Si, U and N animals relative to N. Right panel shows representative section of EMSA for all groups, top arrow indicates the NF-kappa B complex, middle arrows indicate other specific bands and lowest arrow indicates free probe. **(B)** Left panel shows OD% for western blot against Lamin B1 for the same samples used in **(A)**. Center panel shows OD% for the relation p65/Lamin B1 for the same samples. Right panel shows representative western blot sections for all groups. Bars indicate mean ± SEM. *P < 0.01.

S and U animals present a hippocampal nuclear activation of NF-kappa B 45 min post-training as previously reported by our lab (Freudenthal et al., 2005). This activation of the transcription factor during the consolidation time-window is in agreement with the behavioral experiments showed in Figure 1; where both S and U animals show retention of two different learnt tasks in one contextual setting. The experiment to evaluate the activation levels of NF-kappa B in the nucleus of hippocampal cells was controlled in the present work including the Si group of animals. We found that the nuclear transcription factor is not activated in the nucleus of hippocampal cells 45 min post-training in Si animals when compared to naïve (ANOVA *F*_(3,58)_ = 6.694; Mean Si = 102.8 ± 7.997 SEM, *n* = 15; Mean *N* = 100 ± 4.765 SEM, *n* = 15) but both S and U animals showed the previously described activation at this time (ANOVA *F*_(3,58)_ = 6.694; *p* < 0.01; Mean *S* = 125.8 ± 7.402 SEM, *n* = 16; Mean *U* = 136.3 ± 6.702 SEM, *n* = 16; Figure 2A). A representative shift for each group is shown (Figure 2A right panel). The nuclear marker Lamin B1 was used as a control for nuclear extract protein amount in SDS-PAGE for the same samples from Figure 2A right panel. No significant differences were found between groups, indicating that the differences observed in the EMSA are specific to different activation levels of NF-kappa B (Figure 2B left panel). The amount of p65 subunit in the nucleus 45 min post-training remains constant within groups relative to Lamin B1 (Figure 2B center panel). Representative western blots for Lamin B1 and p65 are shown (Figure 2B right panel). This result supports the idea that both S and U groups are undergoing the process of consolidation of contextual associative learning task where NF-kappa B is playing a critical role. In contrast, since no activation of NF-kappa B was observed in the Si group, we might speculate that this group might not be forming hippocampal dependent context associative memories.

### Hippocampal Injections of Sulfasalazine Immediately Post-Training Impair Long-Term Memory Consolidation for Both S and U Animals

**Figure 3:**
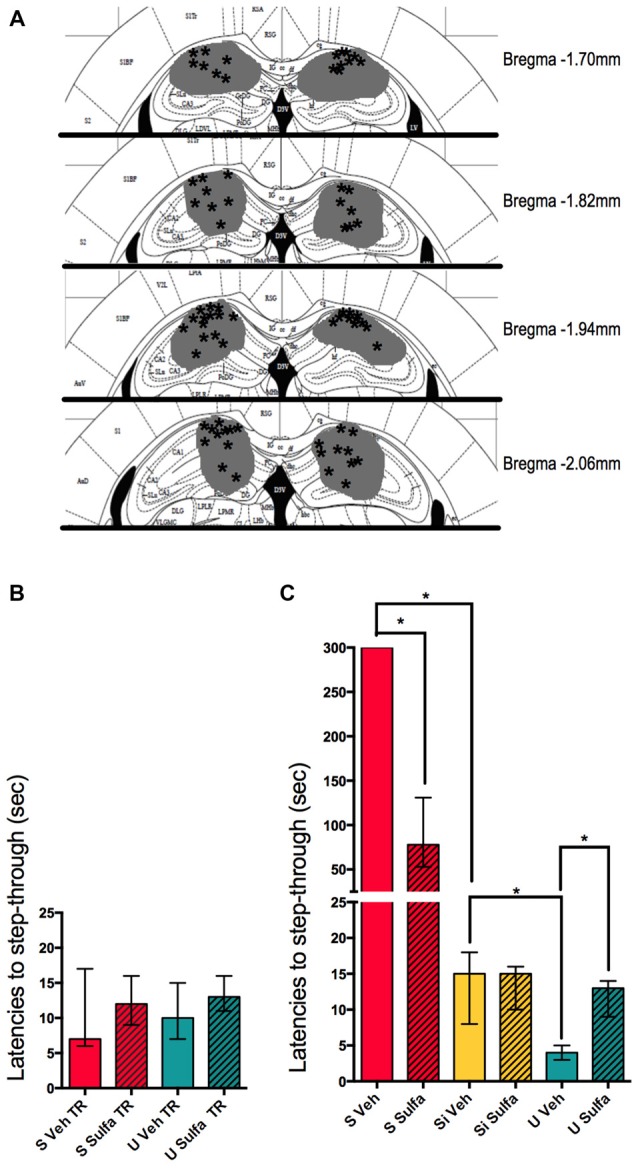
**Intra-hippocampal injections of sulfasalazine impair aversive and appetitive memory. (A)** Mouse atlas sections corresponding to the targeted distance from Bregma are shown. Gray represents the maximum area reached by India ink. Asterisks indicate tip of infusion cannula. **(B)** Latencies to step-through during training for animals in the S, and U groups prior to injection with either Sulfasalazine (Sulfa) or Vehicle (Veh). **(C)** Latencies to step-through during testing 48 h post-training for S, Si and U groups injected with either Sulfa or Veh immediately post-training. Bars show medians with interquartile ranges. *P < 0.01.

To compare the role of the NF-kappa B pathway in the hippocampus, these types of learning animals were injected with the NF-kappa B pathway inhibitor Sulfasalazine. Immediately after training either 1 μg/hippocampus Sulfasalazine in DMSO solution or DMSO (vehicle) was injected (Figure 3A). The groups of animals showed no differences in their step-through latencies during training, before the injection of either drug or vehicle (Figure 3B). When comparing only vehicle injected animals, the same pattern evidenced in Figure 1A is observed, S Veh animals show higher latencies to step-through than Si Veh animals (Mann-Whitney *U* = 0.0; *p* < 0.01; Median S Veh = 300, *n* = 7; Median Si Veh = 15, *n* = 7); while U Veh animals show lower latencies to step-though when compared to Si animals (Mann-Whitney *U* = 1.0; *p* < 0.01, Median Si Veh = 15, *n* = 7; Median U Veh = 4, *n* = 7), evidencing retention in both S Veh and U Veh groups (Kruskal-Wallis statistic *H*_(5,36)_ = 35.41, *p* < 0.01; Figure 3C). Si animals injected with Sulfasalazine (Si Sulfa) show no significant differences in latencies to step-through during testing when compared to Si animals injected with vehicle (Si Veh; Mann-Whitney *U* = 22.5; *p* = 0.845, Median Si Veh = 15, *n* = 7; Median Si Sulfa = 15, *n* = 7) (Kruskal-Wallis statistic *H*_(5,36)_ = 35.41, *p* < 0.01; Figure 3C). As described previously in our lab (Boccia et al., 2007), S animals injected with sulfasalazine (S Sulfa) showed lower latencies to step-through than S animals injected with vehicle (S Veh; Mann-Whitney *U* = 0.0; *p* < 0.01; Median S Veh = 300, *n* = 7; Median S Sulfa = 78, *n* = 7); showing that Sulfasalazine injected post-training intra-hippocampus impairs memory consolidation for S animals (Kruskal-Wallis statistic *H*_(5,36)_ = 35.41, *p* < 0.01; Figure 3C). Interestingly, U animals injected with sulfasalazine (U Sulfa) showed higher latencies to step-through than U animals injected with vehicle (U Veh; Mann-Whitney *U* = 0.5; *p* < 0.01; Median U Veh = 4, *n* = 7; Median U Sulfa = 13, *n* = 7); indicating that Sulfasalazine is causing memory impairment in the U group of animals as well as the S group of animals (Kruskal-Wallis statistic *H*_(5,36)_ = 35.41, *p* < 0.01; Figure 3C). This experiment shows that both S and U animals form hippocampal dependent memories that can be impaired when injecting an inhibitor of the NF-kappa B pathway such as Sulfasalazine. Therefore the NF-kappa B pathway in the hippocampus is necessary for the consolidation of contextual-associative memories both for S and U tasks.

### Amygdala Injections of Sulfasalazine Immediately Post-Training Impair Long-Term-Memory Consolidation for Both S and U Animals

**Figure 4:**
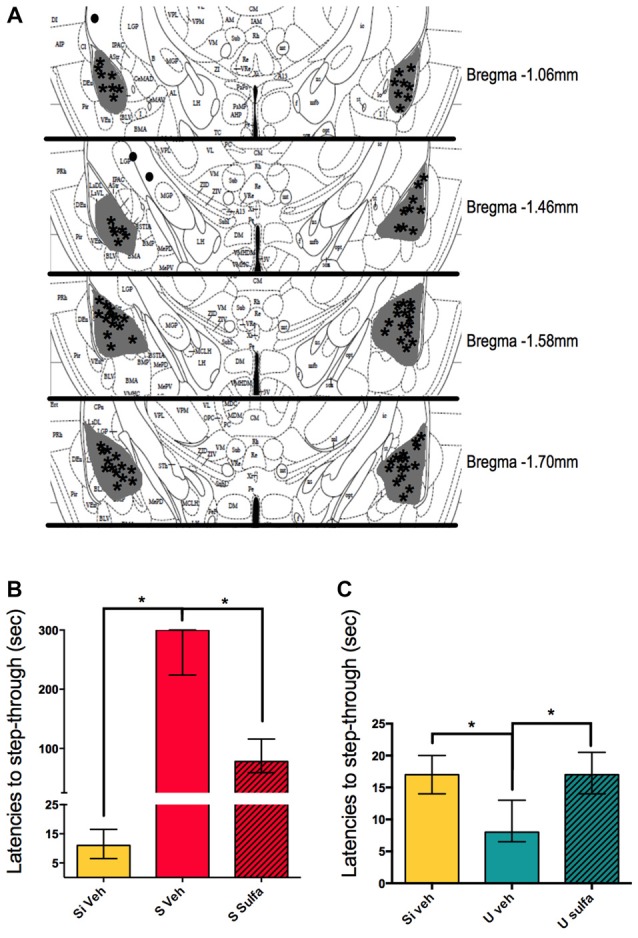
**Intra-amygdala injections of sulfasalazine impair aversive and appetitive memory. (A)** Mouse atlas sections corresponding to the targeted distance from Bregma are shown. Gray represents the maximum area reached by India ink. Asterisks indicate tip of infusion cannula. Black dots indicate injections in animals that did not reach targeted area and thus were discarded from analysis. **(B)** Latencies to step-through during testing for groups Si Veh, S Veh and S Sulfa are shown. **(C)** Latencies to step-through during testing for groups Si Veh, U Veh and U Sulfa are shown. Bars show medians with interquartile ranges. *P < 0.01.

To further study the mechanisms involved in the consolidation of contextual-associative memory we targeted the amygdala. We decided to test the NF-kappa B dependent involvement of this area in both S and U versions of the one trial task. For this, all animals were cannulated bilaterally to reach the amygdala and either 1 μg/amygdala Sulfasalazine or Vehicle was injected immediately post-training (Figure 4A). The Si animals injected with vehicle were used as the behavioral control group. The S Veh animals showed longer latencies to step-through during testing when compared with the Si Veh group (Mann-Whitney *U* = 0.0; *p* < 0.01, Median Si Veh = 11, *n* = 9; Median S Veh = 300, *n* = 9) and this retention was impaired in the S Sulfa group when compared to S Veh (Mann-Whitney *U* = 6.0; *p* < 0.01; Median S Veh = 300, *n* = 9; Median S Sulfa = 78, *n* = 7) (Kruskal-Wallis statistic *H*_(2,22)_ = 20.11, *p* < 0.01; Figure 4B). The amygdala plays a central role in the consolidation of memory in the IA task, and this role is dependent of the NF-kappa B pathway. For the Un-shocked version of the task Sulfasalazine also affected the latencies to step-through. The U Veh animals show lower latencies to step-through than Si Veh animals (Mann-Whitney *U* = 10.0; *p* < 0.01; Median Si Veh = 17, *n* = 11; Median U Veh = 8, *n* = 9). As in the Shock version of the task, this memory is also impaired when animals are injected with Sulfasalazine. The U Veh animals show lower latencies to step-through than U Sulfa animals (Mann-Whitney *U* = 7.5; *p* < 0.01, Median U Veh = 8, *n* = 9; Median U Sulfa = 17, *n* = 9) (Kruskal-Wallis statistic *H*_(2,25)_ = 35.41, *p* < 0.01; Figure 4C). When compared to vehicle, Sulfasalazine had no effect on latencies to step-through during testing for the Si group (Supplementary Figure S1A). In mice, the consolidation of both Shocked and Un-shocked memories seems to be dependent of the NF-kappa B pathway of the amygdala. This evidence supports that the amygdala plays a central role in the consolidation of memory regardless of the valence of the stimulus.

### Nucleus Accumbens Injections of Sulfasalazine Immediately Post-Training Impair Long-Term Memory Consolidation for Both S and U Animals

**Figure 5:**
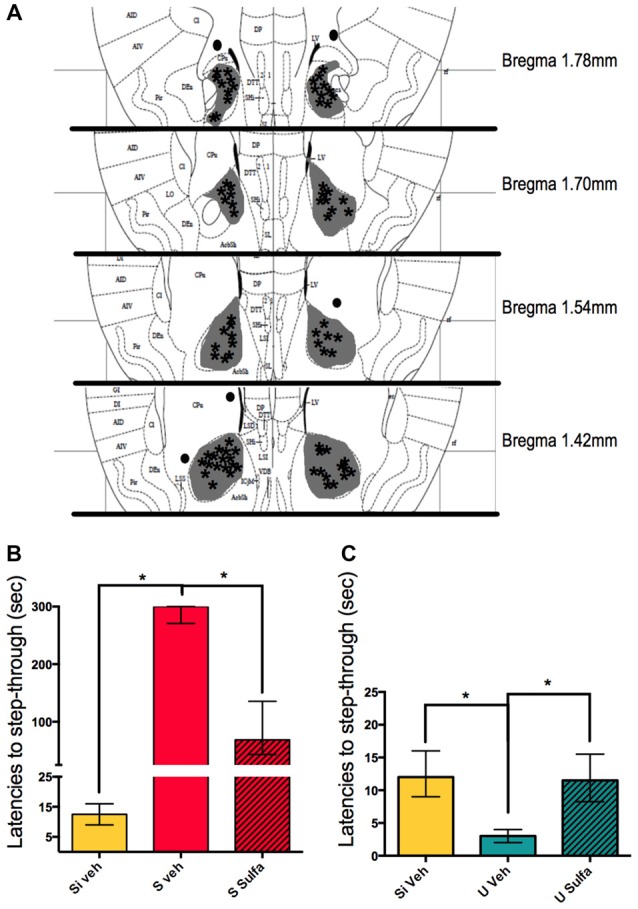
**Intra-nucleus accumbens injections of sulfasalazine impair aversive and appetitive memory. (A)** Mouse atlas sections corresponding to the targeted distance from Bregma are shown. Gray represents the maximum area reached by India ink. Asterisks indicate tip of infusion cannula. Black dots indicate injections in animals that did not reach targeted area and thus were discarded from analysis. **(B)** Latencies to step-through during testing for groups Si Veh, S Veh and S Sulfa are shown. **(C)** Latencies to step-through during testing for groups Si Veh, U Veh and U Sulfa are shown. Bars show medians with interquartile ranges. *P < 0.01.

In pursuit of understanding further which areas are involved in the formation of different valence memories we decided to study the nucleus accumbens. Animals were cannulated bilaterally to reach the nucleus accumbens as described in materials and methods and 1 μg/nucleus accumbens of Sulfasalazine or vehicle was injected on each side immediately post-training (Figure 5A). The S Veh animals showed significantly higher latencies to step-through than Si Veh animals (Mann-Whitney *U* = 0.0; *p* < 0.01; Median Si Veh = 12.5, *n* = 10; Median S Veh = 300, *n* = 10). The S Sulfa animals showed shorter latencies to step-through than S Veh animals (Mann-Whitney *U* = 9.0; *p* < 0.01; Median S Veh = 300, *n* = 10; Median S Sulfa = 68.5, *n* = 10) (Kruskal-Wallis statistic *H*_(2,27)_ = 24.36, *p* < 0.01; Figure 5B). This result implies that the NF-kappa B pathway in the nucleus accumbens is necessary for the consolidation on long-term Shocked memories. On the other hand we showed that the NF-kappa B pathway in the nucleus accumbens is also necessary for long-term consolidation of Un-shocked memories. The U Veh animals showed shorter step-through latencies than Si Veh animals (Mann-Whitney *U* = 1.0; *p* < 0.01; Median U Veh = 3, *n* = 7; Median Si Veh = 12, *n* = 7) and U Sulfa animals (Mann-Whitney *U* = 0.0; *p* < 0.01; Median U Veh = 3, *n* = 7 Median U Sulfa = 11.5, *n* = 8) (Kruskal-Wallis statistic *H*_(2,19)_ = 13.26, *p* < 0.01; Figure 5C). Sulfasalazine had no effect on latencies to step-through during testing for the Si group when compared to vehicle (Supplementary Figure S1B). Therefore sulfasalazine impairs long-term memory consolidation of both Shocked and Un-shocked memories when injected intra-nucleus accumbens. The NF-kappa B pathway in the nucleus accumbens is involved in the consolidation of memory regardless of the valence of the stimulus.

### Sulfasalazine Injections Impair Memory Consolidation Only When the Targeted Areas Are Related to Retention of the Task

**Figure 6:**
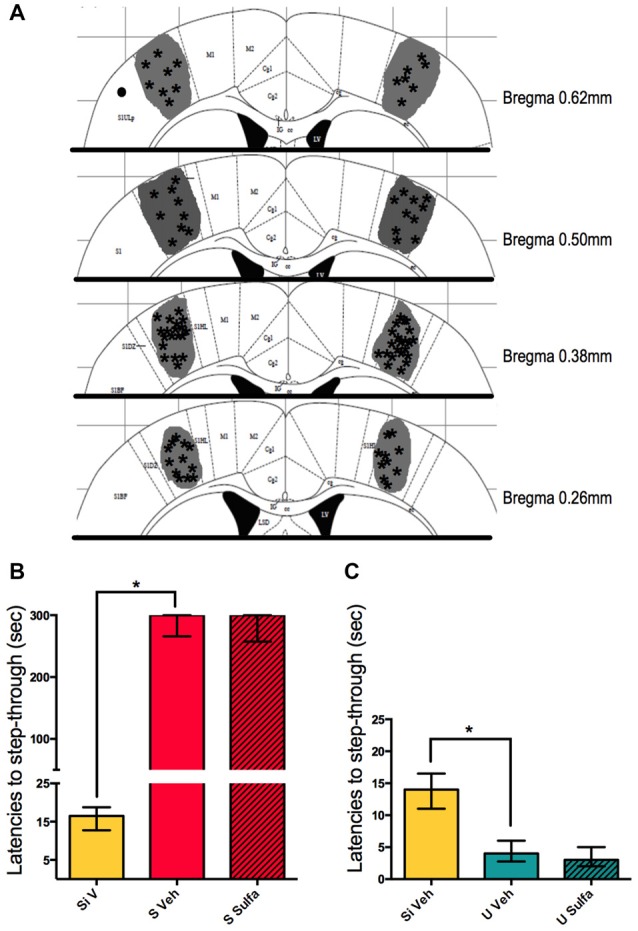
**Intra-primary somatosensory cortex, forelimb region (S1FL) injections of sulfasalazine do not affect aversive and appetitive memory. (A)** Mouse atlas sections corresponding to the targeted distance from Bregma are shown. Gray represents the maximum area reached by India ink. Asterisks indicate tip of infusion cannula. Black dots indicate injections in animals that did not reach targeted area and thus were discarded from analysis. **(B)** Latencies to step-through during testing for groups Si Veh, S Veh and S Sulfa are shown. **(C)** Latencies to step-through during testing for groups Si Veh, U Veh and U Sulfa are shown. Bars show medians with interquartile ranges. *P < 0.01.

An experiment was carried on to evaluate the region specificity of the Sulfasalazine injections. The primary somatosensory cortex, forelimb region (S1FL) was chosen as an area not expected to be related to the memory consolidation of this task. The animals were cannulated bilaterally as described in materials and methods, and either vehicle or sulfasalazine (1 μg/cortex) was injected (Figure 6A). The S Veh animals showed memory retention when compared to Si Veh animals (Mann-Whitney *U* = 0.0; *p* < 0.01; Median Si Veh = 16.5, *n* = 8; Median S Veh = 300, *n* = 9) and S Sulfa animals showed no memory impairment when compared to the S Veh group (Mann-Whitney *U* = 35.0; *p* = 0.9294; Median S Veh = 300, *n* = 9; Median S Sulfa = 300, *n* = 8) (Kruskal-Wallis statistic *H*_(2,22)_ = 18.25, *p* < 0.01; Figure 6B). The U Veh animals showed lower latencies to step-through when compared to Si Veh animals (Mann-Whitney *U* = 2.0; *p* < 0.01; Median Si Veh = 14, *n* = 9; Median U Veh = 4, *n* = 10). The U Sulfa animals showed similar latencies to step-through than U Veh group, implying there was no memory impairment effect of Sulfasalazine (Mann-Whitney *U* = 36.5; *p* = 0.5081; Median U Veh = 4, *n* = 10; Median U Sulfa = 3, *n* = 9) (Kruskal-Wallis statistic *H*_(2,26)_ = 16.92, *p* < 0.01; Figure 6C). This supports the findings that the NF-kappa B pathway inhibition affects memory retention only in areas that are relevant to the consolidation of the Shocked and Un-shocketd tasks.

## Discussion and Conclusion

In the IA paradigm a widely used behavioral control group is a set of animals that do not receive the electric shock after step-through, this group is denominated the Un-shocked group (U). Nevertheless this carries the biased thought that because the animal is not receiving an electric shock it is not forming a contextual associative memory. This idea overlooks the animal’s natural thigmotaxis and negative phototaxis response. Mice tend to move towards walls and corners, thigmotaxis, and away from bright or illuminated areas, negative phototaxis. These are adaptive responses to avoid predators or aversive environments (Pompl et al., 1999; Bonsignore et al., 2008). In this context, the illuminated platform is perceived as an uncomfortable situation for the mice and the dark compartment without the electric shock as a relief from this situation. We argue that another necessary control is the Immediate Shock group (Si) that was described by Wiltgen et al. (2001) in which animals are placed into the dark compartment directly from the top and have no access to the illuminated platform; and immediately receive a foot shock before being returned to their home cage. We found that both S and U animals show different responses during testing in comparison to training, and that the response of Si animals is comparable to either S or U training (Figure 1). The interesting feature of this experiment is that the contextual information perceived by all groups during testing is the same, yet the associations made during training shape the behavioral output: increasing the latencies in S animals and decreasing them in U animals. Therefore we suggest that these one trial contextual tasks can be excellent tools to study and compare the appetitive component of relief and aversive memories.

The hippocampus has been described to be involved in context associative memories (Smith and Bulkin, 2014) and the dynamics of the activation of NF-kappa B during memory consolidation in this and other paradigms has been widely reported (Freudenthal et al., 2005; de la Fuente et al., 2014, 2015; Salles et al., 2015; Zalcman et al., 2015). Specifically, the transcription factor NF-kappa B has been described to be necessary for memory consolidation in several species such as crabs and mice (Freudenthal and Romano, 2000; Freudenthal et al., 2005). Our lab showed that in this IA task in mice, both S and U animals present a hippocampal nuclear activation of the transcription factor 45 min post-training in comparison with Naïve animals (Freudenthal et al., 2005). This activation of the transcription factor during the consolidation window is in agreement with the behavioral experiments showed in Figure 1 where both S and U animals show retention of two different learnt tasks in one contextual setting. In the present work the experiment to evaluate the activation levels of NF-kappa B in the nucleus of hippocampal cells included the Si group of animals. We found that the transcription factor is not activated in the nucleus of hippocampal cells 45 min post-training in Si animals when compared to naïve (Figure 2). The amount of p65 subunit remains constant within groups, implying that the differences observed in S and U groups (Figure 2A) are due to the activation of the NF-kappa B dimer and not to an increase of the total amount of p65 protein. Here we show that U animals show decreased latencies to step-through on the 1st day of re-exposure to context and that on the seventh consecutive day of re-training these latencies remain low. It has been published previously that animals pre-exposed to the context for seven consecutive days (as was done in the experiment of Figure 1C) do not present activation of NF-kappa B 45 min on the seventh consecutive day of U re-training. On the other hand, if animals are given an electric shock on the 7th day of re-exposure (and thus a new association is formed) they showed increased nuclear NF-kappa B activation 45 min post-training (Boccia et al., 2007). These results suggest that for both S and U groups, hippocampal NF-kappa B is involved in the consolidation of contextual associative memories. The lack of NF-kappa B activation on Si animals further supports the idea this group is not forming a contextual association to the electric shock while both S and U animals are forming contextual memories of opposite valence.

NF-kappa B has not only been described to be involved in memory consolidation and reconsolidation but furthermore it has been described to be necessary for this process (Freudenthal et al., 2005; Boccia et al., 2007). Nevertheless the role of NF-kappa B in the hippocampus for appetitive memories in this type of context has not been thoroughly described yet. Therefore we used Sulfasalazine intra-hippocampal injections to study the nature of the relief memory of U animals and compare it with that of S animals. We found that for the NF-kappa B pathway in the hippocampus is necessary for both the Un-shocked and Shocked contextual tasks (Figure 3). This is not an unexpected result since the contextual information received by both groups is the same, and as stated before, one of the most relevant functions of the hippocampus is to process this contextual information. The NF-kappa B pathway is needed for the consolidation of both these contextual memories in this brain area.

We took advantage of having this contextual paradigm that initially, during training, requires the same action from all animals but has opposing behavioral outputs during testing; depending on the training. Therefore we decided to use the training paradigm in both its Shocked and Un-shocked versions to investigate other areas of the brain involved in the consolidation of each type of memory. The amygdala has been widely suggested as an area involved in the processing of the emotional value of memories. Historically it has been mostly associated with the processing of aversive memories (Hermans et al., 2014; Namburi et al., 2016). Nevertheless, there have been recent reports that suggest a role of this area in the processing of appetitive memories (Fernando et al., 2013). We found that both for U and S groups the NF-kappa B pathway in the amygdala is necessary for memory consolidation (Figure 4). This adds to the evidence that the amygdala is not only involved in the processing of aversive memories but that it is also involved in the processing of other biologically relevant events, of varied valence. The dark compartment becomes a positive stimulus for the animal because it provides relief from the illuminated platform (Pompl et al., 1999; Niewalda et al., 2015).

The nucleus accumbens is an area extensively described as being involved in the formation of appetitive, reward and relief-like memories (Bruning et al., 2016; Kahl and Fendt, 2016; Namburi et al., 2016); and there are studies that show the requirement of protein synthesis during this process in the nucleus accumbens (Bruning et al., 2016). Nevertheless, there are some reports that suggest a role of this area in aversive and fear memory formation (Martinez et al., 2008). Therefore we thought it would be interesting to study both versions of the task inhibiting the NF-kappa B pathway in this area. Interestingly, our results show that memory consolidation also depends on the NF-kappa B pathway in the nucleus accumbens for both Shocked and Un-shocked tasks (Figure 5). It is interesting to note that while the Un-shocked situation may have a relief component during training, the Shocked situation does not possess such component. The results of these experiments suggest the nucleus accumbens is needed for both types of memories. Furthermore, we show that NF-kappa B pathway in this area is necessary for memory consolidation in both tasks.

Inhibiting the NF-kappa B pathway only impairs memory consolidation in areas involved in memory processes. An injection of Sulfasalazine in the somatosensory cortex S1FL region (which has not been related to memory consolidation processes, Boccia et al., 2007), did not alter retention in either S or U animals (Figure 6). Furthermore, Sulfasalazine injections in amygdala and nucleus accumbens in the Si animals had no effect on latencies to step-through when comparing with vehicle on testing day (Supplementary Figure S1).

All the data showed in the present work adds to the evidence that suggest that areas like the nucleus accumbens and the amygdala are involved in the processing of memories of different valence. We suggest these tasks with the S, U and Si groups as an excellent model to study the pathways and mechanisms to compare memories of opposing valences. While there is some evidence of the involvement of the NF-kappa B pathway in the consolidation of fear memories in the amygdala (Si et al., 2012), it is the first time that this pathway is shown to be needed in this area for the consolidation of relief memories. Although there is one study that found the NF-kappa B pathway is activated during the re-activation of conditioned place preference memories in the nucleus accumbens (Ye et al., 2014), there haven’t been studies of the involvement of the NF-kappa B pathway during memory consolidation in this area. Nevertheless, NF-kappa B has been described to be involved in the structural changes in the nucleus accumbens after chronic cocaine use (Russo et al., 2009). Therefore this is the first time that the involvement of the NF-kappa B pathway during memory consolidation in the nucleus accumbens is described. We propose that these same brain areas and the NF-kappa B molecular pathway are involved in the consolidation of relief and aversive memories in this tasks. Further studies are needed to find the specific consolidation mechanisms that lead to these different behavioral outputs. One idea is that this information lies within the identity of the population of neurons within these areas that take part in the consolidation of these memories of different valence.

The present study lays valuable groundwork for the study of appetitive and aversive memory consolidation pathways. It proposes the tasks as functional models for such studies and describes for the first time the involvement of the NF-kappa B pathway in appetitive and aversive memory consolidation in the hippocampus, amygdala and nucleus accumbens.

## Author Contributions

AS, MCK, MB, MMB and RF, performed the experiments. AS and RF wrote the manuscript. MCK, MMB and AR helped correct the manuscript.

## Funding

This work was funded by: Agencia Nacional de Promoción Científica y Tecnológica (PICT 2013-1657, RF; PICT 2013-0375, MMB). Universidad de Buenos Aires UBACyT 2014-2017 (20020130200283BA, RF; 20020130100881BA, MMB). Consejo Nacional de Investigaciones Científicas y Técnicas PIP CONICET 2014-2016 (11220130100519CO RF and AR).

## Conflict of Interest Statement

The authors declare that the research was conducted in the absence of any commercial or financial relationships that could be construed as a potential conflict of interest.

## Supplementary Material

FIGURE S1 **Intra-amygdala and Intra-nucleus accumbens injections of sulfasalazine do not have an effect on latencies to step-through for the Si group during testing.** **(A)** Latencies to step-through during testing for groups Si Veh and Si Sulfa injected in the amygdala are shown. **(B)** Latencies to step-through during testing for groups Si Veh and Si Sulfa injected in the nucleus accumbens are shown. Bars show medians with interquartile ranges. No significant differences were found between Si Veh and Si Sulfa groups for either area. **(C)** Mouse atlas sections corresponding to the targeted distance from Bregma are shown for amigdala (left panel) and nucleus accumbens (right panel). Gray represents the maximum area reached by India ink. Asterisks indicate tip of infusion cannula.
