# Supplementary figures and images for: Requirement of NF-kappa B Activation in Different Mice Brain Areas during Long-Term Memory Consolidation in Two Contextual One-Trial Tasks with Opposing Valences
Source: Front Mol Neurosci. 2017 Apr 7;10:104. doi: 10.3389/fnmol.2017.00104 (PMC5383659; doi:10.3389/fnmol.2017.00104)

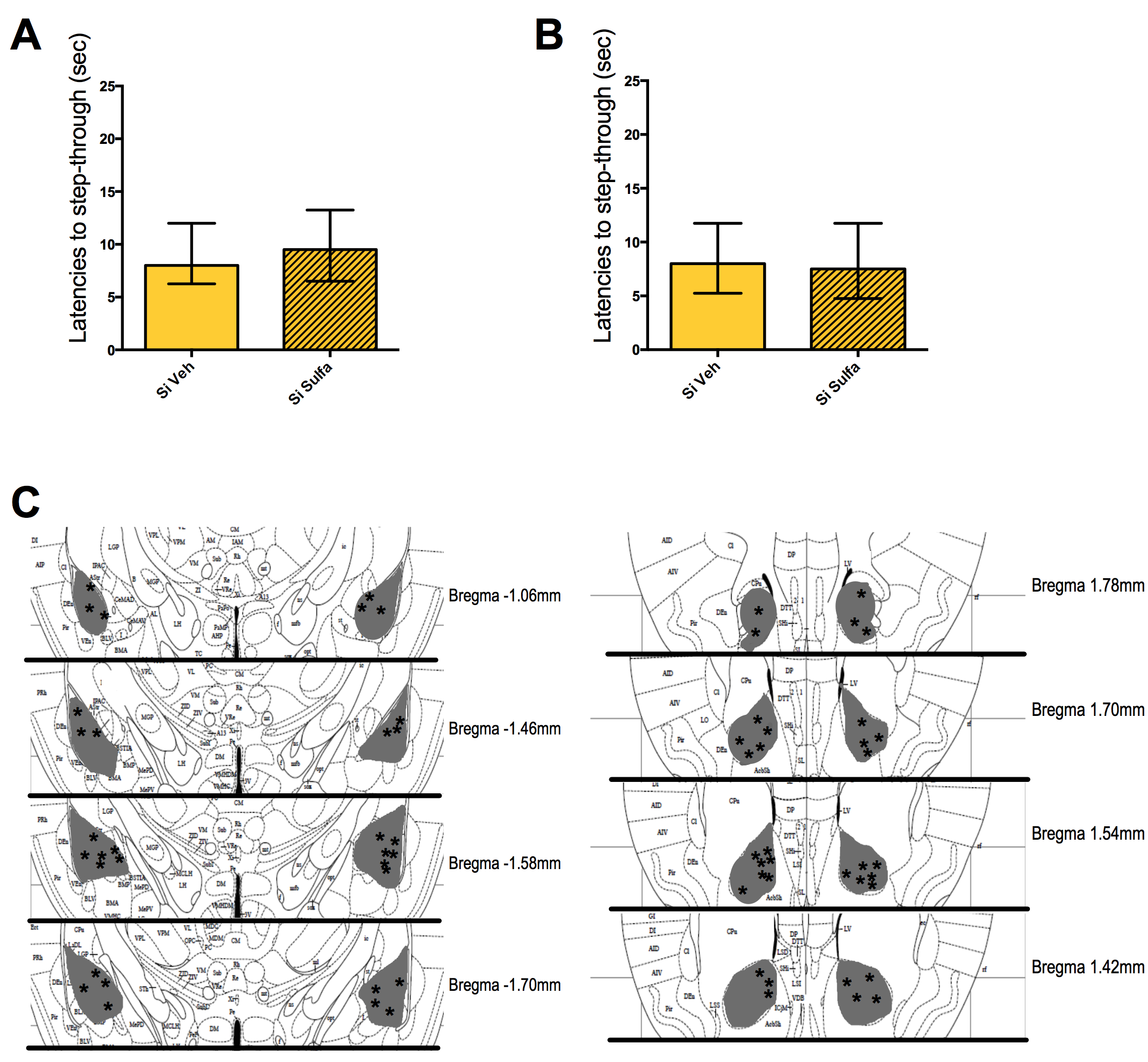

Supplement: FIGURE S1 — Intra-amygdala and Intra-nucleus accumbens injections of sulfasalazine do not have an effect on latencies to step-through for the Si group during testing. (A) Latencies to step-through during testing for groups Si Veh and Si Sulfa injected in the amygdala are shown. (B) Latencies to step-through during testing for groups Si Veh and Si Sulfa injected in the nucleus accumbens are shown. Bars show medians with interquartile ranges. No significant differences were found between Si Veh and Si Sulfa groups for either area. (C) Mouse atlas sections corresponding to the targeted distance from Bregma are shown for amigdala (left panel) and nucleus accumbens (right panel). Gray represents the maximum area reached by India ink. Asterisks indicate tip of infusion cannula. [file Image_1.tiff]
